# Medical and Physical Intelligence

**Published:** 1825-12

**Authors:** 


### MEDICAL AND PHYSICAL INTELLIGENCE.

### MORBID ANATOMY.

1. *Cancer of the Heart.*—M. LEGALAS relates the case of a cancer of the heart: the subject was a child, eleven years of age, who, after suffering from the symptoms of pleuritis and pericarditis, recovered for about a year; afterwards was seized with ascites, and died suddenly. On opening the body, the pericardium was found adherent to the heart, which was two-thirds larger than ordinary; the whole of the right ventricle, and part of the left, being converted into a lardaceous substance, of the nature of the soft cerebriform cancer; the auricles, and the septum cordis, only remaining sound. In the arachnoid membrane there was a little serum; an abundant effusion in the left pleura and abdomen; and a great quantity of mucus in the air-passages. M. Legallas remarks, that these disorders always exist in those cases where syncope, or a sudden cessation of the action of the heart or the circulation, causes death. During life, this child gave no indications of the great alteration found in the heart: he was easily fatigued, sometimes suffered from vertigo, and slept heavily; but his education, and his pursuits as a school-boy, were not interrupted. (*Archives Gen.* Sept.)

2. *Singular Ossification of the Heart.*—M. CHOMEL describes a singular instance of this kind, observed in a patient who died of peritonitis, following a disease of the liver. The heart at its base was surrounded by an osseous band, half a line in thickness, two inches broad in front of the right auricle, but only one inch in the rest of its extent. This band, which seemed to have become developed from the internal face of the pericardium, gave origin to a number of white and reddish bundles, of a somewhat fleshy appearance, which terminated on one side in the pericardium, and on the external surface of the heart, which was otherwise healthy. (*Ibid.*)

### PATHOLOGY.

3. *Case of Inflammation of the Tongue.*—Cases of idiopathic inflammation of the tongue so rarely occur, that our readers will probably be interested in a slight notice of that which fell under the care of Dr. G. It was the first example of the disease he had ever seen. The patient was first seen on the 4th of November last, at which time inflammatory and rheumatic affections of the throat and parotid glands were very common. He was thirty-four years of age, and of a strong constitution. Soon after rising in the morning, he was attacked with severe shivering, which lasted for about an hour, and was then succeeded by considerable heat of the body, which was accompanied by a peculiar weight and immovability of the root of the tongue. The patient, when first visited, complained of much heat; his face was red, the eyes watery; the breathing was rapid, but deep; the pulse hard and quick. He suffered much from a very distressing thirst; and, in addition to the weight and want of power of moving the tongue, he felt a dull pain at the back part of the organ. He swallowed and spoke with difficulty; had much headache, and was very restless. The tongue was clean, very dry, and red, and could not be projected from the mouth: it was not swoln; it appeared to the patient as heavy as if a lump of lead had been in the mouth, and the mere effort to put it out caused great pain and distress. The throat, the amygdalæ, the uvula, and in short all the adjacent parts, were free from any inflammatory affection.—A gargle of decoct. altheæ and nitre, with a grain of tartar stibiat., was used. Barley-water, with simple oxymel, was recommended to be kept occasionally in the mouth; and an opening enema was ordered.

On the following morning the patient was much worse, and the nature of the case was no longer doubtful: it was evidently an instance of *Glossitis.* The tongue was now much swollen, dry, and hard as a board, of a red colour, and excessively painful. It almost entirely filled the mouth, which was half opened, and could not be closed. The patient could not speak, and, to his great torment, could only partially allay the burning heat and great thirst, by swallowing with much difficulty a teaspoonful of fluid. The inspiration was now difficult, and the anxiety very great; the pulse rapid, full, and hard. Twelve ounces of blood were taken from the arm; warm and emollient vapours were applied to the mouth, and bland liquids taken often, and in the smallest quantities.

In the evening, the symptoms were still increased in severity, although the pulse was not so full or hard as in the morning. The blood was buffed; but a trifling and temporary benefit had, however, been produced by the bleeding. The tongue was now projected from the mouth: it continued dry, red, and excessively painful. The jaws could not be moved. The difficulty of breathing was increased; and the patient, who was tormented with the heat of the parts and the constant thirst, could no longer take any fluid. The anxiety was great, and the brain sympathised in the general distress. Fifteen leeches were applied under the chin, and a blister applied over the bites, which was ordered to remain on until it had produced a complete effect. Every quarter of an hour, a little decoction of mallows, with nitre, was injected into the throat, by means of a syringe with a long point. The glyster was repeated. Cold applications of nitre and sal-ammoniac were made to the tongue. The vapour was continued every two hours.

On the 6th, it was found that the patient had passed a sleepless night, and had been very restless. Towards the morning, the fever and heat abated. Deglutition was still impeded, but the thirst was less distressing. From the corners of the mouth a tough slime was discharged. On both edges of the tongue, which still remained in other respects as before, were two white and moist streaks. The treatment was continued as before. In the evening he was much better. A copious flow of saliva from the mouth diminished the distress of the patient considerably. The symptoms continued to amend gradually, and by the 14th the patient was able to renew his customary occupation. (GRAFE *und* WALTHER's *Journal für Chirurgie*, band 7, heft 2.)

4. *Suppuration of the Internal Ear, with Loss of the Bones, without Injury to the Sense of Hearing.*—Rosalie W———, a child of four years old, with evident disposition to scrofula, was attacked, on the 12th July, 1823, with severe fever and pains of the neck. On the following day, a scarlet eruption made its appearance. The patient appeared to be insensible, was very drowsy, and occasionally screamed out. On the fourth day, without any external appearances which attracted attention, a considerable purulent discharge was seen to issue from the right ear, which gradually increased, but did not appear in any manner to influence the previous constitutional affections. The child lay until the ninth day in almost a comatose state. Both day and night she screamed violently at intervals, and tossed herself about. From this period the fever gradually declined: she became more sensible, no longer screamed, and gradually recovered from her serious attack. The discharge from the ear, however, continued, notwithstanding various injections which were employed. It became highly offensive; and, in the meatus and ictorius, was now perceived a red substance, similar to a polypus.

During the recovery of the little patient from the febrile disease, it was observed that the right side of the face was fuller than the left, and whenever she was cheerful, and laughed or spoke quickly, the face was drawn towards the left side. The right side of the face was apparently paralysed. It was presumed that this paralytic condition was the consequence of pressure upon the nervus facialis, in its passage through the foramen stylo-mastoideum. Soon after, at intervals of from ten to twelve days, all the bones of the ear came away: first, the malleus; secondly, the stapes; thirdly, the incus. Some weeks after, a considerable spongy fragment was discharged. The expectation that the paralysis of the face would be relieved when the destroyed parts had entirely come away, was not realised. To the gratification and surprise, however, of all parties, the sense of hearing had not suffered in the smallest degree. As a confirmation of the truth of this statement, the child was shown to M. GRAEFE, who was himself convinced of the facts. *(Ibid.)*

5. *Case of Hydrophobia.*—Rebilliard, a man forty-eight years of age, a labourer of Parigny, returning from Chalons on the 2d January, 1824, and having arrived at the entrance of his village about eight o'clock in the evening, perceived that he was followed by a wolf: he struck at the animal, but the blow glanced aside, when the wolf sprung upon him, threw him on the ground, and bit his hand. The next day, about two o'clock, he was visited by M. LEPINE, who found him in the following state:—The fore-finger of the left hand was the only part which had been bitten: the third phalanx was divided in the middle, and the remainder of the finger was only attached by a slender shred of skin. The wound was very painful, and he had passed the night in great agitation. The finger was amputated at the junction of the last phalanx with the metacarpal bone; and Rebilliard bore the operation very patiently, being in no anxiety about his condition: he persuaded himself that the animal was not mad, though it had bit many dogs during the night, and devoured part of them, as was proved by its vomiting up some portions of their bodies before it expired. The body of the wolf, when examined, presented nothing particular, except that on the back and sides there were some spots deprived of hair, and the skin in these places was covered with white scales.—Some spoonsful of a calming drink were administered during the day.

On the 7th of January, the wound presented nothing particular. The patient was going on well, except that his sleep was disturbed, and that he imagined the animal was going to devour him. He could not get rid of this idea; to which, however, he appeared to attach but little importance.

On the 16th, Rebilliard went to Chalons to see M. Lepine. His wound was then healing, his appetite good, and he was following his usual occupation: this continued up to the 3d of February.

The next day, (that is, thirty-two from the accident,) he began to feel generally indisposed, but he could not express his feelings: he was anxious about his condition; his head was confused, and in pain; he was tormented night and day by the sight of the animal that bit him.

On the 6th, the wound had changed its aspect: it was swollen, the edges were raised, and discharged a brownish serum. The patient refused food, and drank half a glass of wine only in the day.—Infusion of orange-flowers; an opiate at night.

On the 7th, the patient was very much agitated; he had convulsive motions; the pulse was small, hard, and wiry; and he complained of great constriction in his throat.

On the 8th, all the symptoms of hydrophobia were present. In the attacks of convulsion, which were brought on by the least contradiction, he uttered the most frightful cries; and in his tranquil moments he was employed in getting rid of a viscid and glutinous saliva, with which his mouth was always filled; but he was enabled to plunge his hands into water, and to wash his face, without experiencing any disagreeable sensation; and yet he was not able to swallow a draught which M. Lepine gave him. He requested, in a moment of calm, to have his veins opened, in order to put an end to his sufferings; and, when opposed in this design, violent convulsions ensued. At length death terminated his sufferings, on the morning of the 9th of February. (*Revue Medicale*, Août.)

6. *Excessive Secretion of Viscid Mucus in the Stomach.*—The stomach of the same subject was in a condition which is often referred to by medical writers, as preventing the operation of medicines. The internal surface was lined with a thick investment of mucus, so tenacious and dense as to appear like an additional coat, and nothing but actual experiment could have convinced me that such a vitiated secretion could be thus fixed on the villous coat. In attempted to remove it, I inverted the stomach, and washed it, first in cold, then in warm, and subsequently with soap and water, but without removing any notable quantity. I next rubbed it between my hands, as if washing a cloth, by which a few flakes were detached, but the greater part of it still adhered. If this condition of stomach frequently occurs during disease, it must be next to impossible that medicines can be administered with any advantage. There was a considerable quantity of ether mixed with the fluids in the stomach, but even this powerful agent might as well have been placed in contact with dead matter, for any effect it could have produced on a surface coated as this was. The same condition is thought to exist in cynanche trachealis, which frequently renders emetics almost entirely unavailing, unless the most powerful and stimulating are administered. In delicate females habitually costive, and leading sedentary lives, I have several times suspected this state of stomach, which rendered the system almost utterly insensible to the presence of medicines. One instance of this kind, which occurred in the practice of my much esteemed friend, Dr. John W. Buckler, of Baltimore, required the most violent emetics at a time when the patient appeared so much prostrated that a slight exertion must destroy her. The emetic, after some very violent efforts, caused the discharge of a thick, lining, mucous substance, resembling an entire coat of the stomach broken into large flakes, and a great improvement in her state of health immediately ensued. The extreme prostration in this case was the consequence of want of nutrition, as the digestive function must have been for some time altogether suspended. It would be of great moment if we could determine with any certainty the condition of system leading to this state of the stomach, which always must indicate the use of emetics, notwithstanding the apparent debility may be truly alarming. *(The Philadelphia Journal, &c.)*

---

### THERAPEUTICS.

7. *Codeate of Morphia.*—M. Ollivier announces that, in concert with M. Orfila, he has tried upon the human subject the effect of this new salt found in opium, by M. Robiquet, and which goes by the above name. In large doses, (forty grains, for example,) it has produced precisely the same effects as the acetate of morphia; but, in small doses, such as half a-grain, or a grain, it has appeared to possess rather stronger sedative powers. M. Andral (fils) says, that in those trials made at La Charité, it has produced results precisely similar to those obtained from the acetate of morphia.

8. *Chloruret of Lime in Burns.*—M. Lisfranc has found, that in these accidents, after making use of emollient cataplasms for two or three days, the chloruret of lime may be employed with success. It was used in the proportion of four or six ounces to a pint of water, and he thinks this medicine will be found equally useful in hospital gangrene. M. LEGALAS observes, that lately in a case of chronic catarrh of the bladder, he was enabled much to diminish, and for a short time altogether to overcome, the odour of the urine, by injecting into the bladder a fluid, to which a certain quantity of the chloruret of lime was added. *(Archives Gen. Sept.)*

9. *Cauterization of the Pustules of the Small-pox.*—M. MEYRAUX has stated, in a memoir read to the Royal Academy, some interesting facts relative to the above subject. Experience has proved to M. MEYRAUX, that cauterization, in order to be beneficial, must be practised the first or second day of the eruption. When employed later, it has not the effect of destroying or preventing the formatiou of the pustules which follow their regular course, and leave the usual marks and excavations in the skin. Many methods of using the caustic have been practised; that most commonly adopted, is to wash the part with a solution of the nitrate of silver; but the author objects to this method, and mentions many inconveniences attending it, the first of which is, that it acts upon the sound, as well as upon the unsound, parts; and the second is still more forcible, for he asserts, that the development of the pustules still goes on under the black crust, or mask, to the same extent as if it had not been employed. The safest method, according to M. MEYRAUX, is to open the pustules one after the other, and to apply to them a piece of the lunar caustic in the shape of a pencil; for the purpose of opening them, a lancet is the best instrument. It is a very curious circumstance, that the passage of the galvanic fluid, which never produces the inflammation of any organ, completely extinguishes the pustules of small-pox. The most efficacious method of producing this almost instantaneous effect, is to make use of a very fine needle, which is to be placed in the pustules, and the voltaic fluid is to be made to penetrate them. As the success of cauterization is subordinate to the general treatment of the patient, none of the usual indications of cure are to be neglected. The author has remarked, that the application of the caustic only becomes useful, when the inflammatory action has been subdued, either in the principal viscera, or in the skin, when the inflammation of that part predominates, for then the general re-action becomes more intense, and the suppuration of those pustules, which have not been destroyed, is more abundant. M. MEYRAUX has proved, that the advantages of this plan may be extended to cases of boils, venereal pustules, the chicken pock, and some other eruptive diseases. *(Ibid.)*

At a very angry discussion on this subject which recently took place at Paris, and at which some of the most eminent practitioners of that metropolis were present, the general impression seems to have been unfavourable to this new method of treating small-pox by caustic applications. —EDITORS.

### METEOROLOGICAL JOURNAL,

By Messrs. WILLIAM HARRIS and Co. 50, Holborn, London.

*From October 20 to November 19, 1825.*

| | MOON. | Rain gauge | THERM. | | | BAROM. | | DE LUC'S HYG. | | WIND. | | ATMOSPHERIC VARIATIONS. | | |
|---|---|---|---|---|---|---|---|---|---|---|---|---|---|---|
| | | | 9 A.M. | MAX. | MIN. | 9 A.M. | 10P.M. | 9 A.M. | 10P.M. | 9 A.M. | 10P.M. | 9 A.M. | 2 P.M. | 10P.M. |
| Oct. | | | | | | | | | | | | | | |
| 20 | | | 42 | 45 | 35 | 29.00 | 29.14 | 75 | 68 | N | NW | Fine | Fine | Fine |
| 21 | | | 40 | 44 | 38 | 29.30 | 29.52 | 66 | 65 | NNW | NW | | | |
| 22 | | | 44 | 50 | 35 | 29.70 | 29.95 | 79 | 75 | N | N | | | |
| 23 | | | 43 | 47 | 45 | 29.99 | 29.98 | 77 | 77 | WSW | W | Foggy | | |
| 24 | | | 47 | 53 | 40 | 29.84 | 29.74 | 78 | 75 | W | NW | | | Rain |
| 25 | | .06 | 45 | 48 | 33 | 29.78 | 29.85 | 77 | 80 | WNW | WNW | Fine | | Fine |
| 26 | ○ | | 39 | 47 | 42 | 29.98 | 29.85 | 72 | 84 | NW | W | | | Cloud. |
| 27 | | | 44 | 46 | 41 | 29.93 | 29.93 | 82 | 77 | NNE | W | | | Fine |
| 28 | | | 52 | 57 | 45 | 29.87 | 29.93 | 85 | 77 | W | WNW | Foggy | | |
| 29 | | | 53 | 57 | 52 | 29.93 | 29.91 | 81 | 82 | WSW | WNW | Fine | | Cloud. |
| 30 | | | 44 | 55 | 45 | 29.85 | 29.71 | 79 | 77 | WSW | WNW | | | Rain |
| 31 | | .32 | 46 | 53 | 46 | 29.88 | 29.95 | 73 | 75 | W | WNW | | | |
| Nov | | | | | | | | | | | | | | |
| 1 | | | 52 | 56 | 45 | 29.81 | 29.60 | 83 | 84 | W | W | Rain | | Rain |
| 2 | | .29 | 46 | 52 | 51 | 29.69 | 29 52 | 75 | 81 | WNW | SW | Fine | | Sleet |
| 3 | ☾ | | 53 | 48 | 35 | 28.89 | 29.04 | 65 | 83 | WSW | WNW | | | Rain |
| 4 | | .23 | 41 | 45 | 34 | 29.44 | 29.76 | 75 | 75 | WNW | N | | | |
| 5 | | .36 | 35 | 45 | 52 | 29.86 | 29.60 | 79 | 85 | WNW | SWva. | | Rain | Sleet |
| 6 | | | 56 | 56 | 38 | 29.08 | 29.15 | 80 | 76 | W | W | | Fine | Fine |
| 7 | | | 40 | 43 | 34 | 29.41 | 29.25 | 72 | 75 | WNW | W | | | Rain |
| 8 | | .39 | 36 | 45 | 40 | 29.30 | 28.95 | 78 | 86 | WSW | SW | | | Foggy |
| 9 | | | 43 | 45 | 37 | 29.01 | 29.08 | 75 | 75 | WSW | WSW | | | |
| 10 | ● | | 41 | 43 | 36 | 28.76 | 28.85 | 96 | 90 | E var. | NNE | Rain | Rain | Rain |
| 11 | | .23 | 40 | 42 | 35 | 29.29 | 29.50 | 81 | 80 | N | N | Cloud. | Fine | Fine |
| 12 | | | 36 | 39 | 30 | 29.72 | 29.83 | 71 | 71 | NNW | NNE | Fine | | |
| 13 | | | 31 | 36 | 36 | 29.84 | 29.80 | 78 | 71 | NNW | NW | | | |
| 14 | | | 42 | 45 | 35 | 29.75 | 29.85 | 84 | 74 | WNW | N | Foggy | | |
| 15 | | | 39 | 43 | 32 | 29.97 | 30.08 | 78 | 75 | N | WNW | Fine | | |
| 16 | | | 35 | 40 | 38 | 30.08 | 30.10 | 77 | 85 | NW | WNW | Foggy | | Cloud. |
| 17 | ☽ | | 40 | 44 | 38 | 30.00 | 29.77 | 90 | 90 | W | WNW | Fine | | |
| 18 | | .13 | 41 | 51 | 40 | 29.87 | 29.67 | 87 | 87 | WSW | WSW | | Rain | Sleet |
| 19 | | | 41 | 45 | 35 | 29.80 | 29.89 | 86 | 80 | WNW | WNW | | Fine | Fine |

The quantity of rain fallen in the month of October, was 2 inches and 70.100ths.

